# The Role of Neuroglial Crosstalk and Synaptic Plasticity-Mediated Central Sensitization in Acupuncture Analgesia

**DOI:** 10.1155/2021/8881557

**Published:** 2021-01-18

**Authors:** Zhongxi Lyu, Yongming Guo, Yinan Gong, Wen Fan, Baomin Dou, Ningcen Li, Shenjun Wang, Yuan Xu, Yangyang Liu, Bo Chen, Yi Guo, Zhifang Xu, Xiaowei Lin

**Affiliations:** ^1^Research Center of Experimental Acupuncture Science, Tianjin University of Traditional Chinese Medicine, Tianjin 301617, China; ^2^School of Acupuncture & Moxibustion and Tuina, Tianjin University of Traditional Chinese Medicine, Tianjin 301617, China; ^3^National Clinical Research Center for Chinese Medicine Acupuncture and Moxibustion, Tianjin 300381, China; ^4^Suzuka University of Medical Science, Suzuka 5100293, Japan; ^5^School of Traditional Chinese Medicine, Tianjin University of Traditional Chinese Medicine, Tianjin 301617, China

## Abstract

Although pain is regarded as a global public health priority, analgesic therapy remains a significant challenge. Pain is a hypersensitivity state caused by peripheral and central sensitization, with the latter considered the culprit for chronic pain. This study summarizes the pathogenesis of central sensitization from the perspective of neuroglial crosstalk and synaptic plasticity and underlines the related analgesic mechanisms of acupuncture. Central sensitization is modulated by the neurotransmitters and neuropeptides involved in the ascending excitatory pathway and the descending pain modulatory system. Acupuncture analgesia is associated with downregulating glutamate in the ascending excitatory pathway and upregulating opioids, 𝛾-aminobutyric acid, norepinephrine, and 5-hydroxytryptamine in the descending pain modulatory system. Furthermore, it is increasingly appreciated that neurotransmitters, cytokines, and chemokines are implicated in neuroglial crosstalk and associated plasticity, thus contributing to central sensitization. Acupuncture produces its analgesic action by inhibiting cytokines, such as interleukin-1*β*, interleukin-6, and tumor necrosis factor-*α*, and upregulating interleukin-10, as well as modulating chemokines and their receptors such as CX3CL1/CX3CR1, CXCL12/CXCR4, CCL2/CCR2, and CXCL1/CXCR2. These factors are regulated by acupuncture through the activation of multiple signaling pathways, including mitogen-activated protein kinase signaling (e.g., the p38, extracellular signal-regulated kinases, and c-Jun-N-terminal kinase pathways), which contribute to the activation of nociceptive neurons. However, the responses of chemokines to acupuncture vary among the types of pain models, acupuncture methods, and stimulation parameters. Thus, the exact mechanisms require future clarification. Taken together, inhibition of central sensitization modulated by neuroglial plasticity is central in acupuncture analgesia, providing a novel insight for the clinical application of acupuncture analgesia.

## 1. Introduction

Pain is mediated by nociceptive nerve fibers and is a physiological alarm response to protect the body by reducing tissue damage from injury [1]. Thirty to fifty percent of patients with pathological pain suffer from anxiety and depression long after wound healing. Both the pain and unpleasant emotions affect their quality of life and cause serious social and economic consequences [2, 3]. However, the solution for chronic pain remains a major challenge throughout the world. Oral drugs are often the first choice, and their usage has expanded exponentially in recent years [4]. Unfortunately, the extensive application of analgesic drugs may result in organ damage and abuse, together with causing serious social problems [5]. For example, long-term overuse of opioids, the most common class of prescribed painkillers, can lead to side effects such as addiction, tolerance, and drowsiness, as well as impaired memory, attention, and judgment. Opioid abuse can also potentially cause respiratory depression [6, 7]. Therefore, a natural analgesic that can also regulate pain-related moods and cognitive disorders is necessary.

Acupuncture therapy is a well-known treatment that originated in China and has been applied in 183 countries and regions all over the world [8, 9]. Long-term clinical practice has proved that acupuncture is an effective treatment to relieve pain. The World Health Organization has recommended acupuncture for more than 30 types of pain conditions, including lumbago, headache, sciatica, and postoperative pain [10]. Referring to the National Guideline Clearinghouse (http://www.guidelines.gov/), there are 49 specific medical recommendations for acupuncture, of which 37 (75.51%) are pain-related diseases [11]. At present, many clinical randomized controlled trials (RCTs) have demonstrated the analgesic effect of acupuncture [12]. As shown in a 16-week RCT, compared with sham acupuncture treatment and awaiting-treatment groups, acupuncture treatment could significantly reduce the incidence of migraine without premonitory migraine, an effect that lasted at least 24 weeks [13]. A meta-analysis by Vickers et al. analyzed the individual data of patients in RCTs with nonspecific musculoskeletal pain, osteoarthritis, shoulder pain, and chronic headache, concluding that acupuncture is effective for the treatment of chronic pain and has long-lasting therapeutic effects [12]. These studies have indicated that acupuncture analgesia is effective and safe and can improve the quality of life of patients suffering from pain.

Pain is caused by tissue damage or similar pathophysiological causes. Pain sensitization plays a key role in pain occurrence and maintenance. Pain sensitization starts from the sensitization of peripheral nerves and involves a series of neuroplastic changes in the spinal cord and brain [14, 15], namely, central sensitization. Neurotransmitters, neurotrophic factors, lipids, and cytokines/chemokines play important roles in the communication between neurons and glial cells in both peripheral and central sensitization [16, 17]. Interestingly, increasing evidence suggests that acupuncture can alleviate central sensitization induced by glial cell-mediated inflammation to achieve analgesia [18, 19]. Therefore, we have summarized the analgesic mechanisms of acupuncture in terms of neuroglial crosstalk and neuroplasticity-mediated central sensitization, highlighting the central neuroimmunological regulating mechanisms and providing novel evidence and insights for the clinical application of acupuncture.

## 2. Methods

### 2.1. Search Strategy

We searched the PubMed database for published studies, from January 2010 to April 2020. The keywords included [“acupuncture” or “electroacupuncture” or “manual acupuncture”] and [“pain” or “analgesia” or “analgesic”]. The language was limited to English. The filter process was done firstly by the website's search engine which initially identified 2888 articles.

### 2.2. Study Selection

**Figure 1:**
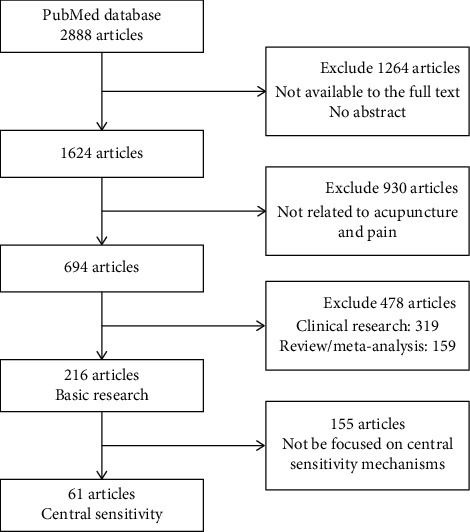
Flow chart of the search processes.

Of these articles, we excluded 1264 articles due to the absence of an abstract or unavailability of the full text, leaving 1624 articles. Hand searching was performed by screening the reference lists of articles that met our inclusion criteria based on the titles and abstracts. Of these, we excluded 930 articles as not related to acupuncture and pain in the titles and abstracts before full-text assessments resulting in 694 articles. These included 216 basic research articles, 319 clinical research articles, and 159 review articles or meta-analyses. The full texts of 216 basic research articles meeting the inclusion criteria were obtained and read carefully. Of these, 155 articles were excluded from 216 basic researches due to not be focused on central sensitivity mechanisms, resulting in 61 articles. A flowchart of the search process is shown in Figure 1.

### 2.3. Data Extraction

Of 61 basic researches on the central mechanism, the information from 35 typical and recently published studies are listed in Table 1 due to similarities in some of the studies, to analyze the central sensitization-related mechanisms of acupuncture analgesia. The study design data were extracted and classified using a predefined data extraction form that designated the pain model type, the intervention (methods, acupoints, acupuncture parameters), and the outcome measures (pain-related behavior, mechanism indexes). The data were extracted mainly by one author and were checked by the other authors.

## 3. Role of Neural-Immune Crosstalk in Peripheral Sensitization

The basic process of pain generation occurs when nociceptors located in the periphery of peptidergic and nonpeptidergic primary nociceptive neurons selectively respond to thermal, mechanical nociception, and irritating chemicals. During this process, related neurons in the dorsal root ganglia depolarize and generate receptor potentials, converting physical or chemical information to electrical information. The electrical information is transmitted along the sensory pathway to the spinal cord and other primary centers. The integrated information from the primary center can be transmitted to higher centers in the brain to form the pain sensation, or to the motor neurons in the anterior horn to produce a reflex [20].

Pain sensitization is a remodeling mechanism of the central and peripheral nociceptive receptors. Continuous stimulation such as tissue and nerve injury remodels the nociceptor to produce pain sensitization, specifically, peripheral sensitization, is indicative of primary hyperalgesia, including peripheral nociceptive receptors and dorsal root ganglion DRG [21]. Neuroimmune interactions are essential for peripheral sensitization [22]. There are two types of peripheral inflammation: tissue and neurogenic inflammation. Tissue damage can cause the local release of histamine, vascular dilatation, increased blood flow, and vascular exudation, as well as the release of various cytokines/chemokines and agglutination factors. The immune cells (including macrophages, mast cells, neutrophils, and lymphocytes) release inflammatory mediators to aggravate pain and, at the same time, release endogenous opioid peptides to inhibit peripheral inflammatory pain [23]. In recent years, cytokines/chemokines have been found to be involved in regulating and sensitizing the excitability of nociceptors. Inflammatory cytokines (interleukin-1*β* (IL-1*β*), IL-6 and tumor necrosis factor-*α* (TNF-*α*)) mediate allodynia (pain resulting from a stimulus that would not normally provoke pain), hyperalgesia (increased response to noxious stimulation), and increasing the expression levels of substance P (SP) and prostaglandin E2 (PGE2) in neurons and glial cells in the DRG. Structurally, chemokines are similar to cytokines, and their receptors are expressed in the DRG. For instance, monocyte chemoattractant protein-1 (CCL2) and its receptor, C-C chemokine receptor type 2 (CCR2), were both increased in the nerve injury model. Moreover, CCL2 can promote the interaction between immune cells and nociceptors, thus playing a key role in inflammatory and neuropathic pain [20]. At the same time, the excitatory effect of cytokines/chemokines on nociceptive neurons is manifested by regulating the opening of ion channels; specifically, the activation of chemokine receptors can regulate the current in voltage-gated calcium channels (VGCCs) [20].

Neurogenic inflammation is caused by chemicals, such as SP and calcitonin gene-related peptide (CGRP), that are released from primary nociceptive neurons [24]. Upon peripheral tissue injury, sustained nociceptive stimulation or inflammation sensitizes the nociceptors. These discharges activate 𝛾-aminobutyric acid (GABA) neurons by acting on *N*-methyl-d-aspartate (NMDA) or non-NMDA receptors in the intermediate neurons of the spinal cord and cause depolarization by acting on adjacent primary afferent terminals. The discharge also produces a dorsal root reflex by retrograde conduction, leading to the resensitization of the injury receptors mediated by neurogenic inflammation and finally inducing paresthesia and allodynia [20]. In addition, the sympathetic nervous system participates in strengthening the neurogenic inflammatory process, and neuroimmune crosstalk plays an important role in peripheral sensitivity.

## 4. Central Sensitization in Pain Transmission and Modulation Pathways Is a Crucial Target for Acupuncture Analgesia

Central sensitization, namely secondary hyperalgesia, including the disbalance of the ascending excitatory pathway and descending pain modulatory system, plays a vital role in the development and maintenance of chronic pain [25]. In the ascending excitatory pathway, amplification of neural signaling within the central nervous system (CNS) produces a state of neuronal hyperactivity and hyperexcitability in the spinal cord and brain, leading to hyperalgesia [26]. This occurs mainly in the lamina I and lamina V neurons of the spinal cord, as well as in medullary reticular formation, thalamus, hypothalamus, cerebellum, amygdala, basal ganglia, hippocampus, cortexes S1 and S2, insula, anterior cingulate cortex (ACC), prefrontal cortex, and some related cortical areas in the parietal and temporal lobes [27–30]. The descending pain modulatory system arises from a number of supraspinal sites, including the midbrain periaqueductal gray (PAG) that projects indirectly to the spinal cord via the rostral ventromedial medulla (RVM), lateral and caudal dorsal reticular nucleus (DRN), and ventrolateral medulla, of which the PAG-RVM system has been most studied [31].

As shown in Table 1, the neuropathic pain models, including pain generated by chronic constriction injury, chemotherapy-induced pain, spinal nerve ligation, and spinal cord injury, have been widely used in the study of central sensitization-mediated acupuncture analgesia. Inflammatory pain induced by the administration of complete Freund's adjuvant also causes central sensitization, including the dual mechanisms of peripheral and central sensitization. Many of these studies have used electroacupuncture (EA) rather than manual acupuncture, and the acupressure points ST36 and SP6 have been most used. Both low frequencies at 2-15 Hz and, alternatively, higher frequencies between 2 Hz and 100 Hz were effective in EA analgesia, providing useful information for maximizing the effects of acupuncture analgesia in clinical settings.

### 4.1. Acupuncture Inhibits the Ascending Excitatory Pathway

As described in Section 1, nociceptive receptors are composed of types A*δ* and C sensory fibers which transduce noxious stimuli from damaged peripheral tissues to the spinal cord. Neuroplasticity in the dorsal horn of the spinal cord assists and enhances pain perception by the nociceptive neurons. Central sensitization includes three processes: activation, sensitization, and modification of the CNS [32]. These are characterized by increases in the excitatory postsynaptic current (EPSC), including windup, long-term potentiation (LTP), and disinhibition. Among these, LTP caused by both high- and low-frequency electrical stimulation, nerve damage, or tissue damage is a process in which a transient synaptic activity can produce a long-lasting increase in synaptic strength. LTP relies on NMDA receptors (NMDARs) and voltage-dependent calcium channels, and results in calcium influx, thereby activating protein kinase A, protein kinase C, and calcium/calmodulin-dependent protein kinase II (CaMKII) [33]. Protein synthesis is necessary for late-phase LTP (L-LTP), and administration of brain-derived neurotrophic factor (BDNF) in the spinal dorsal horn and the hippocampus can induce L-LTP of C-fiber potentials [34]. Disinhibition includes a reduction of GABAergic and/or glycinergic inhibition and an enhancement of A*β* and C fiber sensitivity. The loss of inhibitory interneurons, impaired storage and/or release of inhibitory neurotransmitters, and impaired postsynaptic receptor activity have been proposed as the mechanisms underlying disinhibition [35]. Finally, the integrated signals travel through the spinal cord to the thalamus, where the location and intensity of the pain is processed. The signal then travels to the classic pain nerve circuit, the cerebral sensory cortex (S1, S2), which distinguishes the location and duration of the pain [36]. Apart from S1 and S2, neuronal activities in the ACC, insular cortex, and amygdala also contribute to various aspects of pain perception, including the experience of discomfort [37].

In terms of the molecular mechanisms, primary nociceptive neurons release glutamate, SP, CGRP, adenosine triphosphate (ATP), and neurokinin-1 (NK1) into the spinal cord. These molecules interact with their receptors, including NMDARs, *α*-amino-3-hydroxy-5-methyl-4-isoxazolepropionic acid receptors (AMPAR), metabotropic glutamate receptors (mGluR), SP receptors (NK1R), calcitonin receptor-like receptors (CRLR), and purinergic receptors (P2X, P2Y) of the spinal nociceptive projection neurons. This induces an influx of Ca^2+^ into the pain neurons of the spinal cord [38], activating calcium-dependent intracellular cascades, inducing NMDAR phosphorylation [39], ultimately resulting in the generation of nociceptive information throughout the spinothalamic pain pathway [40, 41]. Thus, these mediators released by nociceptive neurons cause neural hyperexcitability and nociceptive transmission.

Glutamate is the most important and widely distributed excitatory neurotransmitter in the CNS and plays a critical role in the ascending excitatory pathway. Glutamate receptors are broadly divided into two groups, metabotropic glutamate receptors (mGluRs), and cation-permeable ionotropic glutamate receptors (iGluRs), which are, in turn, subdivided into NMDAR, Kainate-type iGluR, and AMPAR. Excitatory glutamatergic transmission plays a crucial role in the onset of chronic pain and is characterized by an increase in glutamate concentrations and receptor stimulation, leading to the transmission of pain messages [77, 78]. Somers et al. investigated the effects and potential mechanisms of transcutaneous electrical nerve stimulation (TENS) on neuropathic pain caused by chronic constriction injury (CCI). The mechanical allodynia was relieved by decreased concentrations of excitatory glutamate in the dorsal horn with a combination of low- and high-frequency TENS [79]. Recent evidence has shown that 2 Hz EA bilateral stimulation on the *Zusanli* (ST36) and *Sanyinjiao* (SP6) acupressure points produced analgesic effects through the downregulation of the *N*-methyl-d-aspartate receptor type 2B (NR2B, NMDAR subunit), inhibiting the transmission of pain messages in a CCI rat model [42]. AMPAR consists of four subunits (GluR1-R4). EA has been demonstrated to prevent phosphorylation of AMPAR, especially the GluR2 subunit, in a Complete Freund's Adjuvant (CFA) model [43, 44]. In addition, the antinociceptive effect of EA is proposed to be related to the recovery of glutamate transporter (GT) expression, which can remove excess glutamate from the synaptic clefts. Both the spinal glutamate-aspartate transporter and GT1, which are mainly distributed in glial cells, were increased after EA treatment in CFA-injected rats as a result of proteasome downregulation [45]. A similar response has also been observed in a neuropathic pain model of spared nerve injury treated with EA [46]. Glutamatergic synaptic transmission is coupled with excess Ca^2+^ entry into projection neurons and results in the activation of the Ca^2+^-dependent enzyme Ca^2+^/calmodulin-dependent protein kinase II (CamKII) and phosphorylation of the NR2B subunit of NMDAR at postsynaptic sites in the ACC, thus modulating visceral pain in a viscerally hypersensitive model [80]. It was reported that EA at ST36 and *Kunlun* (BL60) could reverse the actions of the calcium voltage-gated channel subunit and calcium voltage-gated channel auxiliary subunit *γ*, thus reducing chronic inflammatory pain (CIP) in the CFA rats [47].

### 4.2. Acupuncture Regulates the Descending Pain Modulatory System

It is now clear that the descending pain modulatory pathway can be both facilitatory and inhibitory, with a dynamic balance between the two functions. When acute pain turns to chronic pain, the descending facilitation function is dominant, leading to enhanced pain sensitization and even “mirror pain,” in which the rostral RVM plays a key role. Descending projections from the RVM, identified as ON and OFF cells, facilitate and inhibit spinal nociceptive transmission, respectively. For example, RVM lesions or functional silencing can prevent pain sensitization induced by nerve injury. Excitatory amino acids, AMPAR, NMDAR, and the BDNF/TrkB signaling pathway are also involved in the process of descending facilitation. Several neurotransmitters (e.g., opioid, GABA, norepinephrine, and 5-hydroxytryptamine (5-HT)) are involved in these descending pathways [20]. It has been demonstrated that acupuncture functions mainly through facilitating the descending inhibitory system to ease pain, while the regulation of acupuncture on the descending facilitation system is poorly understood. Limited results have suggested that EA relieves inflammatory pain via inhibiting the activation of p38 mitogen-activated protein kinases (MAPK) in the central descending facilitation system [48]. In addition, EA potentiates the descending inhibitory control of 5-HT in the medulla via cannabinoid receptors on GABAergic but not glutamatergic neurons, thus inhibiting knee osteoarthritis pain [49].

#### 4.2.1. Regulation of Opioids and Their Receptors by Acupuncture

Opioids and their receptors help reduce the excitatory transmitter release in the midbrain descending pathway, especially in the PAG-RVM system [81]. Kissiwaa et al. found that opioid receptors, mainly the *μ* opioid receptors (MORs), could inhibit glutamate release at synapses in the amygdala [82]. In addition, the analgesic capacity of MORs in the PAG was negatively regulated by glutamate-binding NMDAR NR1 subunits [83]. Until now, the activation of endogenous opioids (e.g., enkephalin, *β*-endorphin, and dynorphin)/opioid receptor (e.g., *μ* and *δ* opioid receptors) is the best-understood mechanism of acupuncture analgesia [50, 84–87]. Although different frequencies of EA can be reversed by the opioid antagonist naloxone, the types of opioids that mediate EA effects vary according to the EA frequency. For example, a single administration of 2 Hz EA at ST36 for 20 minutes can alleviate hyperalgesia during ethanol withdrawal through the mediation of MORs in the lateral habenula (LHb), an epithalamic structure rich in MORs. Activation of MORs may inhibit the release of glutamate in the LHb, which can block the descending nociceptive signal from the LHb to the PAG, thus reducing pain [50]. Besides MORs, 10 Hz EA at *Huantiao* (GB30) can significantly relieve paclitaxel-induced mechanical allodynia and hyperalgesia via the *δ* or *κ* opioid receptors [51].

#### 4.2.2. Regulation of Nonopioid Neurotransmitters by Acupuncture

*(1) GABAergic Inhibitory Interneuron Network*. GABA is an important inhibitory neurotransmitter that is involved in the reduction of pain sensation through presynaptic inhibition [88]. The imbalance between excitatory glutamatergic and inhibitory GABAergic transmission, particularly decreased inhibition of GABAergic synaptic transmission in the spinal pain circuit, has been considered to underlie the development of central sensitization [23, 27, 52, 89, 90]. It was also found that RVM GABAergic neurons could facilitate mechanical pain by inhibiting dorsal horn enkephalinergic/GABAergic interneurons [91]. EA may initiate analgesia by increasing GABA expression on the descending pain modulatory pathways in the PAG [52]. Furthermore, the GABA receptor, a ligand-gated ionotropic receptor, is located mainly in postsynaptic neurons and contributes to the initiation of fast synaptic inhibition, thus being the major target for producing sedation [92]. EA at the bilateral L4 and L6 of *Hua Tuo Jia Ji* points (EX-B2) [53] or ST36 and *Yanglingquan* (GB34) points can significantly reduce neuropathic pain by increasing the GABA receptors in the spinal cord [54]. Moreover, EA can support the GABAergic system by reducing the rate of GABA reuptake. GABA transporter-1 (GAT1), the dominant neuronal GABA transporter, controls GABA concentrations, and EA was found to reduce GAT1 activity via activating the *δ*-opioid receptor in the PAG [93]. Taken together, acupuncture can modulate the GABAergic system by increasing GABA expression, activating the GABA receptor, and inhibiting GABA reuptake.

*(2) Norepinephrine*. Norepinephrine is released from descending projection into the dorsal horn and helps to evoke an antinociceptive effect. The depletion of spinal norepinephrine caused mechanical hypersensitivity in a dose-dependent manner in a chronic pain rat model [94]. Studies have clearly shown that norepinephrine is involved in acupuncture analgesia. A previous report by Choi et al. demonstrated that EA stimulation (2 mA, 2 Hz, 30 min, once every two days) at bilateral ST36 could significantly diminish paclitaxel-induced neuropathic pain. Further studies have shown that the antinociceptive effect of EA was mediated by spinal descending adrenergic pathways through the activation of the *α*2- and *β*-spinal adrenoceptors in mice [55, 56]. Consistent with these results, studies have shown that EA-induced analgesia in a rat model of an ankle sprain is mediated mainly by suppressing dorsal horn neuron activity through *α*-adrenoceptors [57]. In addition, studies have shown that the analgesic effect of 10-Hz-EA was regulated by the spinal *α*2a-adrenoceptor, compared to the *α*2b-adrenoceptor in a CFA-induced inflammatory pain model [58]. It has also been found that intrathecal injection of desipramine, a selective noradrenaline uptake inhibitor, increased the availability of spinal noradrenaline and prolonged the antinociception effect of EA [59].

*(3) 5-Hydroxytryptamine*. 5-HT (also called serotonin) arises largely from the RVM to the spinal cord and exerts biphasic modulation in the descending facilitation and inhibitory pathways. The facilitatory and inhibitory influence on the spinal processing of nociceptive information were mediated through recruitment of RVM ON or OFF cells, respectively, and depended on acute or chronic pain states and the type of receptor acted upon [60, 95]. A low dose of 5-HT produces facilitation of fast EPSCs in the spinal cord, while a high dose of 5-HT induces inhibition of AMPA/kainite-receptor-mediated EPSCs [88, 96]. Numerous studies have shown the involvement of the 5-HT and 5-HT receptors in acupuncture analgesia effects [60, 97–99]. EA at 100 Hz, but not 2, 50, or 2/100 Hz, was effective in alleviating the pain-depression dyad and upregulating 5-HT in the DRN of reserpine-injected rats [60]. In a CFA-inflammatory pain model, 10 Hz EA inhibited thermal hyperalgesia through the activation of spinal 5-hydroxytryptamine 1A receptors (5-HT1AR) but not 5-HT2BR, 5-HT2CR, or 5-HT3R [58, 61]. Consistent with these results, EA activated spinal 5-HT1AR to alleviate both allodynia and hyperalgesia in CIP rats [62]. In an osteoarthritis model, the effectiveness and mechanisms of EA were focused on the involvement of spinal 5-HT2A/2C receptors [63]. Taken together, these data suggest that spinal 5-HT and its receptors are involved in acupuncture analgesia but the effects differ according to both animal models and EA frequencies.

### 4.3. Role of the Tripartite Synapse in Acupuncture Analgesia

#### 4.3.1. Neuroglial Crosstalk Regulates the Neural Plasticity

Vladimir Parpura first proposed the concept of the “tripartite synapse” in 1994 [100]. This concept added glial cells (astrocytes and microglia) to the original classic pre- and postsynaptic neuronal cells, thus emphasizing the important roles of glial cells in synaptic transmission and regulation. It is noteworthy that recent studies have shown that central sensitization driven by neuroinflammation in the CNS is accompanied by glial activation, including the activation of astrocytes, microglia, and oligodendrocytes in the spinal cord and brain [101]. Lau and coworkers found that L5 spinal nerve ligation (SNL) caused the upregulation of cyclooxygenase-1 (COX-1) and COX-2 in spinal microglial and neuronal cells; these enzymes are involved in the production of prostaglandins (PGs) via arachidonic acid pathways and, in turn, lead to the development of neuropathic pain [102]. Functional imaging also showed elevated cerebral levels of the translocator protein, a marker of glial activation, in patients with chronic low back pain [103]. Therefore, chronic pain may also be the result of “gliopathy” [104].

It is increasingly clear that neural plasticity is regulated by neuroglial crosstalk. After peripheral tissue or nerve damage, neurotransmitters such as glutamate, SP, and CGRP released from nociceptive primary afferent fibers in the dorsal horn not only cause intense high-frequency activation of the neural postsynaptic receptors and amplification of the postsynaptic current but also interact with their corresponding receptors on microglia and astrocytes [105–107], activating the voltage-dependent calcium channel and inducing calcium entry into the neuron. Glia-derived substances are termed gliotransmitters and include cytokines (IL-1, IL-6, and TNF-*α*), chemokines (CCL2 [108] and CXCL1 [109]), and inflammatory mediators (e.g., bradykinin, PGs, and nitric oxide). These chemicals promote an inflammatory environment [101] and act as chemical mediators to amplify neuroglial reactivity in a paracrine manner, favoring the elevation of these mediators in the dorsal horn of the spinal cord [110]. As an example, TNF-*α* acting at the TNF-*α* receptor 1 (TNFR1) functions in the development of pain by facilitating excitatory synaptic signaling in the acute phases after nerve injury in CCI mice compared with sham control mice [111]. TNF-*α* contributes to neural plasticity through mechanisms involving the downregulation of GTs, upregulation of the glutamate concentration in the synapse, and phosphorylation of NMDAR. TNF-*α* subsequently promotes the facilitation of excitatory synaptic transmission and downregulates the expression of GABA receptors, thus reducing the inhibition of excitatory transmission [112]. Chemokines can also regulate the interactions of neurons and glial cells, e.g., CCL2 derived from astrocytes can “talk to” neurons by regulating neuronal activity via c-Jun-N-terminal kinase (JNK) MAPK [108]. These mediators produced by active glial cells contribute to neural plasticity. During the process, the activated glia can release prostaglandin, BDNF, nitric oxide, and other neuroactive substances; reduce the inhibitory effects of GABA; and upregulate the expression of NMDARs, thereby increasing nerve excitability and maintaining neuropathic pain [22, 113]. Moreover, modification of glial cell numbers in the brain is likely correlated with the emotional experience of pain, as well as mood disorders such as depression and anxiety [114]. Once activated, the glial cells in the spinal cord release cytokines that provide positive feedback, further enhancing excitatory synaptic transmission.

#### 4.3.2. Inhibition of Glial Activity by Acupuncture

It has been shown that in the neuropathic pain model induced by SNL, the inhibition of spinal microglia and astrocytes mediates the immediate and long-term EA analgesia, respectively [64]. It has also been reported that the analgesic efficacy of EA might be related to the modulation of microglial and astrocyte activation [115–117]. Acupuncture has been reported to suppress signal transduction pathways and key molecules, including p38 MAPK, extracellular regulated kinases (ERK), and JNK, in microglial and astrocyte activation in pain processing [65, 66, 118–120]. Previous results observed that the acupuncture analgesic effect is related to spinal cytokines and neurotrophic factors released by glial cells [67, 121, 122]. Repeated EA treatment at the bilateral ST36 and GB34 points once a day can relieve chronic pain and suppress the elevated mRNA expression of TNF*α* and IL-1*β* in the spinal cord of CCI rats [67]. EA (2/100 Hz, 2 mA) for five consecutive days can significantly increase the mechanical threshold and thermal latency after CFA injection. This could be partially associated with the suppression of proinflammatory cytokines (e.g., TNF-*α* and IL-1*β*) and the stimulation of IL-10 in the spinal cord. IL-10, produced by the spinal cord, is the key anti-inflammatory cytokine for relieving both inflammatory pain [68, 69] and neuropathic pain [70]. Paclitaxel significantly activates both microglia and astrocytes and increases the expression of inflammatory cytokines (IL-1*β* and TNF-*α*) in the lumbar spinal cord. EA treatment (10 Hz, 1 mA) at the bilateral ST36 point in rats suppressed the expression of inflammatory cytokines through the downregulation of the TLR4/NF-*κ*B pathway as well as suppressing activated microglia and astrocytes [71]. Besides TLR4, increased expression of TLR2 in the spinal cord and thalamus was also reportedly suppressed by EA in a CFA model [72]. Therefore, the downregulation of gliotransmitters by acupuncture can prevent the activation of neuroglial crosstalk, thus contributing to the easing of chronic pain. However, the evidence on how acupuncture modulates glial cells to inhibit excitatory synaptic transmission is still incomplete.

### 4.4. Role of Chemokines and Their Receptors in Acupuncture Analgesia

#### 4.4.1. Introduction to the Chemokine System

Chemokines, 8-12 kDa secreted proteins, constitute the largest family of cytokines. According to the number and spacing of cysteines, chemokines consist of two major families, CC (CC_1-28_) and CXC (CXC_1-16_) chemokines, as well as two minor families, XC (XC_1-2_) and CX3C (CX3CL1) chemokines [123]. All chemokines bind to the members of a family of seven transmembrane-spanning heterotrimeric G protein-coupled receptors (GPCRs). Chemokines are regarded as important mediators of inflammation and help to control the positioning and migratory patterns of immune cells. Immune cell residence in primary (T cells, B cells), secondary (lymph nodes, spleen, Peyer's patches), and tertiary lymphoid organs are under the fine control of this complex system of approximately 50 endogenous chemokines [123]. The development of T cells in the thymus depends on the interaction of epithelial-derived CCL21, CCL25, and CXCL12 with CCR7, CCR9, and CXCR4, respectively, expressed on T cell progenitors [124]. In contrast to the thymus, the homeostasis and development of immune cells in the bone marrow seems to be governed by the opposing forces of interactions between CXCL12/CXCR4 and CCL2/CCR2 [123]. Chemokines, considered as regulators of peripheral immune cell transport, are capable of inducing the migration of T, NK cells, dendritic cells, and/or macrophages [125]. There has been growing recognition that the chemokine system orchestrates immune cell migration (e.g., macrophages [126] and lymphocytes [127]) into the DRG and CNS. This plays a critical role in the central sensitization in the early initiation of pain perception [128]. Besides, chemokines and their receptors expressed by neurons and glial cells in the CNS have been shown to mediate neuroglial communication and nociceptive signal transmission at different anatomical locations, including nerves, the DRG, spinal cord, and brain [129, 130].

#### 4.4.2. Acupuncture Analgesia via Regulation Chemokine and Their Receptors

Concomitant with the increasing use of acupuncture to alleviate pain, attention has been paid in recent years to the mechanism of acupuncture analgesia from the perspective of chemokines.

*(1) CX3CL1/CX3CR1*. CX3CL1 is specifically expressed in neurons and binds to the CX3CR1 receptor on the microglial cell membrane [125], activating the MAPK pathway. MAPKs play important roles in information transmission between neurons and glial cells as well as the genesis of pain hypersensitivity induced by CX3CL1/CX3CR1 [131, 132]. Gao et al. reported that peripheral injury to the primary afferent nociceptive neurons caused the release of CX3CL1 into the spinal cord, which activated the production of TNF*α* in a p38 MAPK-dependent mechanism in microglia. In turn, the activation of TNF-*α* regulates CCL2 expression in astrocytes in a JNK MAPK-dependent manner. CCL2 subsequently activates central neurons through CCR2, eventually leading to neuropathic pain [133]. Neutralizing antibodies to CX3CL1 or CX3CR1 could attenuate mechanical hyperalgesia in neuropathic pain models [134, 135]. It has been demonstrated that neuron-microglia interactions are mediated by purinergic receptors, and, subsequently, by CX3CL1/CX3CR1. The purinergic P2X7R/CX3CL1/CX3CR1 pathway, following intracellular phosphorylation of microglial p38 MAPK [136] which subsequently stimulates the release of IL-6 and IL-1*β* [137], plays a key role in nociceptive signal transmission [138].

In a rat neck-incision pain model, Gao et al. found that two sessions of EA at *Futu* (LI18), *Hegu* (LI4), *Neiguan* (PC6), or ST36, GB34 could significantly relieve thermal pain, followed by the downregulation of ATP/P2X7R/CX3CL1/CX3CR1 signaling and suppression of its downstream p38 MAPK pathway in the upper cervical spinal cord after three sessions. Thereby, EA suppressed ATP/P2X7R/CX3CL1/CX3CR1/p38 MAPK-induced neuroglial crosstalk in pain processing [73]. The results are consistent with the reports by Li that 2 Hz EA at ST36 for 30 mins reduced the overexpression of CX3CL1 other than CX3CR1 in the spinal cord of the CFA model rats. EA inhibited the activation of neuronal and microglial cells and decreased p38 MAPK signaling and the downstream proinflammatory cytokines IL-1, IL-6, and TNF-*α*. They also found that EA did not inhibit the expression of p38 MAPK but inhibited its phosphorylation [74]. Slightly different from their results, the report by Li et al. [75] demonstrated that mechanical allodynia and thermal pain induced hyperalgesia by paw incision was significantly suppressed by acupuncture-combined anesthesia (ACA). However, the analgesic effect of ACA was not apparent in CX3CR1 knockout mice and was also blocked when a neutralizing antibody to CX3CR1 was intrathecally injected 1 h before ACA in C57BL/6J mice, suggesting that CX3CR1 in microglia is not only involved in postincision pain, but also in ACA-induced analgesia.

*(2) CXCL12/CXCR4*. CXCL12 and CXCR4 are, respectively, expressed in neurons and glial cells in the central nervous system [139], and the CXCL12/CXCR4 activation leads to increased pain sensitivity in the spinal cord. A study by Luo et al. demonstrated that CXCL12 and CXCR4 are upregulated in the spinal cord dorsal horn in chronic postischemic pain (CPIP) mice. Intrathecal blocking of CXCR4 improved mechanical allodynia, suggesting an important role of spinal CXCL12/CXCR4 signaling in ameliorating the pain response [140]. Hu et al. found that EA exerted an analgesic effect on the same rat model of CPIP by suppressing the overexpression of CXCL12/CXCR4 in the spinal cord dorsal horn. Furthermore, they found that EA can effectively inhibit excessive activation of glial cells in the spinal cord and markedly reduce downstream ERK pathway activation, thus reducing the central sensitization and exerting an analgesic effect [76].

*(3) CCL2/CCR2*. CCL2 is expressed in spinal astrocytes and induces neuronal activation via CCR2 to increase excitatory synaptic transmission (astrocyte-to-neuron signaling), contributing to central sensitization and neuropathic pain development. It has been shown that CCL2/CCR2 was upregulated in the spinal cord via the JNK pathway after SNL [108]. CCL2 can also rapidly increase NMDA-induced current and spontaneous EPSCs [108] or inhibit GABA-induced currents [141] in dorsal horn neurons, all of which are critically involved in the maintenance of pain. Furthermore, spinal administration of CCL2 induced thermal hyperalgesia via activating the spinal transient receptor potentia1 vanilloid 1 (TRPV1) receptors [142]. Lee et al. demonstrated acupuncture at *Shuigou* (GV26) and GB34 significantly alleviated both mechanical allodynia and thermal hyperalgesia after spinal cord injury at L4-L5. It is noteworthy that acupuncture inhibited the astrocyte expression of CCL2, which is known to be mediated through the JNK pathway and contributes to excitatory synaptic transmission. Apart from the findings on CCL2, Lee also showed that JNK-dependent CCL4 and CCL20 expression was significantly decreased by acupuncture treatment [66].

*(4) CXCL1/CXCR2*. CXCL1 and CXCR2 are expressed in astrocytes and neurons, respectively, in the spinal cord, and CXCL1/CXCR2 in the lumbar spinal cord has been demonstrated to play key roles in pain processing. The application of CXCL1 in the spinal cord acted on CXCR2, inducing the expression of phosphorylated ERK and cAMP-response element-binding protein, c-fos, and COX-2 in the spinal cord neurons and leading to the subsequent maintenance of neuropathic pain [109]. Similarly, Cao and coworkers showed that astrocytes were activated after inflammation and released CXCL1 in the spinal cord, which could then act on CXCR2 to induce ERK activation, synaptic transmission, and COX-2 expression in dorsal horn neurons, ultimately contributing to the pathogenesis of CFA-induced inflammatory pain [143]. In addition, Xu et al. found that astrocyte-secreted CXCL1 activated spinal cord dorsal horn neurons to express CXCR2 in cancer pain models [144]. Taken together, CXCL1/CXCR2 is involved in the development of neuropathic, inflammatory, and cancer pain.

However, it has also been demonstrated that CXCL1/CXCR2 is involved in pain relief. Previously, a study by Cao et al. demonstrated intrathecal administration of recombinant CXCL1 in wild-type mice significantly reduced spinal nerve L5 transection- (L5Tx-) induced mechanical hypersensitivity. Due to CXCL1's chemotaxis, it is capable of associating with the increased number of opioid peptides produced by infiltrating neutrophils in the lumbar spinal cord [145]. Similarly, Guo and his colleagues showed that bone marrow stromal cell-induced monocytes secrete CXCL1 which could cross the blood-brain barrier and contribute to pain relief. The CXCL1/CXCR2 signaling triggers opioid release by activation of central *μ*-opioid receptors in the RVM [146]. Furthermore, we have observed a novel function of acupuncture-derived CXCL1 in the CFA rat model. In our unpublished data, it was found that acupuncture at ST36 could induce high CXCL1 levels in CFA rat serum. CXCL1 neutralizing antibodies reduced the acupuncture analgesic effect by 20%. Our results demonstrated that acupuncture-derived CXCL1 can induce spinal cord CXCR2 desensitization, blocking COX2 production in the spinal cord. These phenomena showed that the same chemokines involved in pain sensitization may also be neuroprotective and promote pain relief under certain conditions.

## 5. Conclusion

**Figure 2:**
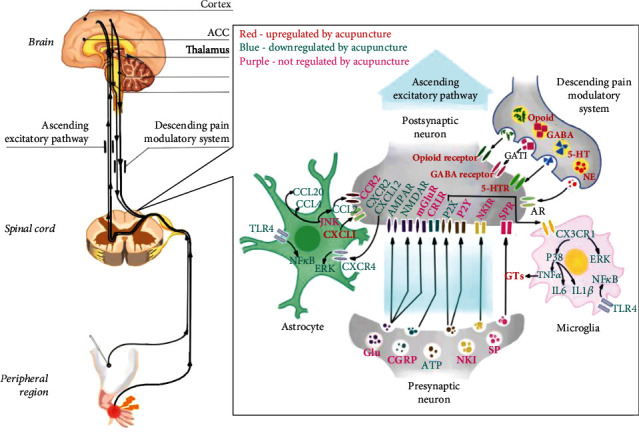
Role of neuroglial crosstalk and synaptic plasticity mediated central sensitization in acupuncture analgesia. The titles of neurotransmitters, neuropeptides, and immune factors are presented in red, blue, and purple, as the figure shows. Factors in red are upregulated by acupuncture, while factors in blue are downregulated by acupuncture. Factors in purple are activated or inhibited in the central sensitization process but are not regulated by acupuncture. ACC: anterior cingulate cortex; PAG: periaqueductal gray; RVM: rostral ventromedial medulla; GABA: 𝛾-aminobutyric acid; 5-HT: 5-hydroxytryptamine; 5-HTR: 5-hydroxytryptamine receptor; NE: norepinephrine; AR: adrenergic receptor; GAT1: GABA transporter 1; Glu: glutamate; AMPAR: *α*-amino-3-hydroxy-5-methyl-4-isoxazolepropionic acid receptor; NMDAR: *N*-methyl-d-aspartate receptor; mGluR: metabotropic glutamate receptors; CGRP: calcitonin gene-related peptide; CRLR: calcitonin receptor-like receptor; ATP: adenosine triphosphate; P2X: P2X receptor; P2Y: P2Y receptor; NK1: neurokinin-1; NK1 receptor: neurokinin-1 receptor; SP: substance P; SPR: substance P receptor; CX3CL1: C-X3-C motif chemokine ligand 1; CX3CR1: C-X3-C chemokine receptor 1; ERK: extracellular signal-regulated kinases; TNF-*α*: tumor necrosis factor-*α*; IL-6: interleukin-6; IL-1*β*: interleukin-1*β*; GT: glutamate transporter; TLR4: toll-like receptor 4; JNK: c-Jun-N-terminal kinase; CCL2: C-C motif chemokine ligand 2; CCL4: C-C motif chemokine ligand 4; CCL20: C-C motif chemokine ligand 20; CCR2: C-C chemokine receptor 2; CXCL1: C-X-C motif chemokine ligand 1; CXCR2: C-X-C chemokine receptor 2; CXCL12: C-X-C motif chemokine ligand 12; CXCR4: C-X-C chemokine receptor 4. Red, promoted by acupuncture; blue, inhibited by acupuncture.

To conclude (as shown in Figure 2), it is well established that pain is a hypersensitivity state caused by peripheral and central sensitization. Central sensitization is modulated by the ascending excitatory pathway and the descending pain modulatory system. With the emergence of the “tripartite synapse” concept, neuroglial cells are considered as active partners of neurons at the synapse and can contribute to central sensitization. It is increasingly appreciated that neurotransmitters (e.g., glutamate, opioid, GABA, norepinephrine, and 5-HT), inflammatory cytokines, and chemokines are implicated in this crosstalk. Acupuncture analgesia involves the ascending excitatory pathway and the descending pain modulatory system through the downregulation of glutamate and upregulation of opioids, GABA, norepinephrine, and 5-HT. Evidence indicates that the occurrence and maintenance of pain are closely related to immune responses, and the role of cytokines/chemokines in mediating neuroglial communication has attracted much attention in recent years. Acupuncture analgesia has also been demonstrated to inhibit cytokines such as IL-1*β*, IL-6, and TNF-*α* and upregulate IL-10, as well as modulating chemokines and their receptors such as CX3CL1/CX3CR1, CXCL12/CXCR4, CCL2/CCR2, and CXCL1/CXCR2. Furthermore, acupuncture has been found to regulate downstream neural MAPK signaling (e.g., p38, ERK, and JNK pathways), which contribute to the activation of nociceptive neurons. However, the responses of chemokines to acupuncture differ between pain model types, acupuncture methods, and parameters, requiring future clarification of the exact mechanisms. Taken together, the inhibition of neuroglial plasticity-mediated central sensitization is one of the critical mechanisms in acupuncture analgesia, contributing to the wider application of acupuncture, alone or in combination with pain medication, in the enhancement of treatment effectiveness and the lowering of pain medication dosages and decreasing the risk of debilitating adverse effects. The acupuncture parameters described in these studies, particularly those of EA stimulation, also provide significant information for maximizing the effect of acupuncture in the clinical setting.

## Figures and Tables

**Table 1:** Central sensitization regulatory mechanism of acupuncture analgesia.

Refs.	Pain model	Intervention methods	Acupoints	Acupuncture parameter	Pain-related behavior	Test site	Biochemical measurements
*4.1 Acupuncture inhibits the ascending excitatory pathway*							

Zhao et al. [42]	CCI	EA	ST36, SP6	2 Hz, 2 mA for 30 mins	Mechanical allodynia	Spinal cord	NR2B↓
Lee et al. [43]	CFA	EA	ST36, SP6	2 Hz, 1 mA for 30 mins	Thermal hyperalgesia	Spinal cord	Phospho-GluR2↓
Han et al. [44]	CFA	EA	ST36, SP6	2-15 Hz, 1 mA for 30 mins	Mechanical allodynia, thermal hyperalgesia	Spinal cord	GluR2 phosphorylation↓
Kim et al. [45]	CFA	EA	ST36, SP6	2 Hz, 1 mA for 30 mins	Thermal hyperalgesia	Spinal cord	GFAP↓, GLAST↑, GLT-1↑
Zeng et al. [46]	SNI	EA	ST36, SP6	2 Hz, 3 mA for 30 mins	Mechanical allodynia	Spinal cord	GLAST↑, GLT-1↑
Zhou et al. [47]	CFA	EA	ST36, BL60	Alternative 2 Hz/120 Hz, 1-2 mA for 30 mins	—	Spinal cord	Calcium voltage-gated channel subunit ↓, calcium voltage-gated channel auxiliary subunit gamma↓

*4.2 Acupuncture regulates the descending pain modulatory system*							

Hu et al. [48]	CFA	EA	ST36, BL60	2 Hz/100 Hz, 1-2 mA for 30 mins	Mechanical allodynia	RVM	p38MAPK↓
Yuan et al. [49]	KOA	EA	Ex-LE4, ST35	2 Hz, 15 Hz or 100 Hz, 1 mA/0.1 mA for 30 mins	Mechanical allodynia, thermal hyperalgesia	vlPAG	Endocannabinoid-CB1R-GABA-5-HT↑
Li et al. [50]	Hyperalgesia during ethanol withdrawal	EA	ST36	2 Hz, 0.2-03 mA for 20 mins	Thermal hyperalgesia	Habenula	MORs↑
Meng et al. [51]	Paclitaxel-induced neuropathic pain	EA	GB30	10 Hz, 2 mA for 30 mins	Mechanical allodynia	Spinal cord	*μ* and *δ* opioid receptor↑
Huang et al. [52]	CCI	EA	GV20, GV14	15 Hz, 1 mA for 20 mins	Mechanical allodynia and thermal hyperalgesia	PAG	GABA_A_↑, GABA↑, GLU↓
Jiang et al. [53]	CCI	EA	EX-B2	2/15 Hz, 2 mA for 30 mins	Mechanical allodynia, thermal hyperalgesia	Spinal cord	GABA_A_↑
Li et al. [54]	CCI	EA	ST36, GB34	Alternative 2 Hz/100 Hz, 1.5 mA for 30 mins	Mechanical allodynia, thermal hyperalgesia	Spinal cord	KCC2↑, GABAA receptor *γ*2↑
Choi et al. [55]	PTX-induced neuropathic pain	EA	ST36	2 Hz, 2 mA for 30 mins	Mechanical allodynia and thermal hyperalgesia	Spinal cord	alpha2- and beta-adrenoceptors↑
Choi et al. [56]	PTX-induced neuropathic pain	BVA	LI11, ST36	—	Mechanical allodynia	Spinal cord	*α*2-adrenoceptor↑
Kim et al. [57]	Ankle sprain pain	EA	SI6	10 Hz, 2 mA for 30 mins	Weight-bearing force on the affected foot	Spinal cord	*α*2-adrenoceptor↑
Zhang et al. [58]	CFA	EA	GB30	10 Hz, 3 mA for 40 mins	Thermal hyperalgesia	Spinal cord	*α*2-ARs↑, 5-HTRs↑
da Silva et al. [59]	Uninjured rats	EA	ST36, SP6	2, 100, or 2/100 Hz, 1.4-1.5 mA for 20 mins	Thermal hyperalgesia	Spinal cord	Norepinephrine, acetylcholine, endogenous opioids, or GABA
Wu et al. [60]	Pain-depression dyad	EA	ST36, SP6	100 Hz, 1.0, 1.5, and 2.0 mA, each for 15 min	Mechanical allodynia	Dorsal raphe nucleus	5-HT↑
Zhang et al. [61]	CFA	EA	GB30	10 Hz, 3 mA, twice for 20 min each	Thermal hyperalgesia	Spinal cord	5-HT↑, 5-HT1AR↑
Zhang et al. [62]	CIP	EA	GB30	10 Hz, 2 mA for 30 mins	Mechanical allodynia and thermal hyperalgesia	Spinal cord	5-HT1AR↑, p-CaMKII↓
Li et al. [63]	Osteoarthritis-induced pain	EA	ST36, GB30	10 Hz, 2 mA for 30 mins	Weight-bearing force on the affected foot	Spinal cord	5-HT2A/2C↑

*4.3 Role of the tripartite synapse in acupuncture analgesia*							

Liang et al. [64]	SNL	EA	ST36, BL60	2, 100, or 2/100 Hz, 1-2 mA for 30 mins	Mechanical allodynia	Spinal cord	GFAP↓, OX-42↓
Choi et al. [65]	SCI	MA	GV26, GB34	Turned at a rate of 2 spins per second for 30 s, and retained for 30 mins	Mechanical allodynia, thermal hyperalgesia	Spinal cord	p38MAPK↓, ERK↓, PGE2↓
Lee et al. [66]	SCI	MA	GV26, GB34	Turned at a rate of two spins per second for 30 s, retained for 30 mins	Mechanical allodynia, thermal hyperalgesia	Spinal cord	JNK↓, CCL2↓, CCL4↓, and CCL20↓
Wang et al. [67]	CCI	EA	ST36, GB34	Alternative 2 Hz/100 Hz, 1 mA for 30 mins	—	Spinal cord	IL-1*𝛽*↓, TNF-*α*↓, BDNF↓, NT3/4↓, NGF↓
Yu et al. [68]	CFA	EA	ST36, SP6	Alternative 2 Hz/100 Hz, 2 mA for 20 mins	Mechanical allodynia, thermal hyperalgesia	Spinal cord	IL-10↑, IL-1*β*↓, NLRP3↓, TNF-*α*↓
Dai et al. [69]	Paw incision pain	EA	SP6, GB34	Alternative 2 Hz/100 Hz, 1-2-3 mA for 30 mins	Mechanical allodynia	Spinal cord	IL-10↑, LTP↓
Ali et al. [70]	SNL	EA	ST36, SP6	2 Hz, 2-3 mA for 20 mins	Mechanical allodynia	Spinal cord	IL-10↑, *β*-endorphin↑
Zhao et al. [71]	PTX-induced neuropathic pain	EA	ST36	10 Hz, 1 mA for 30 mins	Mechanical allodynia	Spinal cord	TMEM119↓, GFAP↓, TLR-4/NF-*κ*b↓, TNF-*α*, and IL-1*β*↓
Hsu et al. [72]	CFA	EA	ST36	2 Hz, 1 mA for 15 mins	Mechanical allodynia, thermal hyperalgesia	Spinal cord/thalamus	TLR2↓, pPI3K↓, pAkt↓, pmTOR↓, pERK↓, pp38↓, pJNK↓, pCREB↓, pNF*κ*B↓, Nav1.7↓, Nav1.8↓

*4.4 Role of chemokines and their receptors in acupuncture analgesia*							

Gao et al. [73]	Neck-incision pain	EA	LI18, LI4, PC6, or ST36, GB34	Alternative 2 Hz/100 Hz, 1 mA, 30 mins	Thermal pain	Spinal cord	ATP↓, P2X7R↓, CX3CL1↓, CX3CR1↓, p38 MAPK proteins↓
Li et al. [74]	CFA	EA	ST36	2 Hz for 30 mins	Mechanical allodynia, thermal hyperalgesia	Spinal cord	CX3CL1↓, p38 MAPK phosphorylation↓, IL-1↓, IL-6↓, and TNF-*α*↓
Li et al. [75]	Paw incision pain	ACA	GB34, SP6	Alternative 2 Hz/100 Hz, 1, 2, 3 mA per 10 mins	Mechanical allodynia, thermal pain	Spinal cord	CX3CR signaling
Hu et al. [76]	CPIP	EA	ST36, BL60	Alternative 2 Hz/100 Hz, 0.5-1.5 mA, 30 mins	Mechanical hyperalgesia	Spinal cord	CXCL12/CXCR4↓, ERK↓

ACA: acupuncture-combined anesthesia; BVA: bee venom acupuncture; CCI: chronic constriction injury; CFA: complete Freund's adjuvant; CIP: chemotherapy-induced pain; CPIP: chronic postischemic pain; EA: electroacupuncture; MA: manual acupuncture; SNL: spinal nerve ligation; SCI: spinal cord injury; TENS: transcutaneous electric nerve stimulation; RVM: rostral ventromedial medulla; PAG: periaqueductal gray; vlPAG: ventral lateral periaqueductal gray.

## Data Availability

The generated or analyzed data used to support the findings of this study are included within the article.

## Abbreviations

RTCs: Randomized controlled trials
IL-1*β*: Interleukin-1*β*
TNF-*α*: Tumor necrosis factor-*α*
SP: Substance P
PGE2: Prostaglandin E2
DRG: Dorsal root ganglion
VGCCs: Voltage-gated calcium channels
CGRP: Calcitonin gene-related peptide
GABA: 𝛾-Aminobutyric acid
NMDA: N-methyl-d-aspartate
CNS: Central nervous system
EPSC: Excitatory postsynaptic current
LTP: Long-term potentiation
NMDARs: N-methyl-d-aspartate receptors
CaMKII: Calcium/calmodulin-dependent protein kinase II
L-LTP: Late-phase LTP
BDNF: Brain-derived neurotrophic factor
ACC: Anterior cingulate cortex
ATP: Adenosine triphosphate
NK1: Neurokinin-1
AMPAR: *α*-Amino-3-hydroxy-5-methyl-4-isoxazolepropionic acid receptors
mGluR: Metabotropic glutamate receptors
SPR: SP receptor
CRLR: Like receptor
NK1R: NK1 receptor
iGluR: *α*-Amino-3-hydroxy-5-methyl-4-isoxazolepropionic acid receptor, ionotropic glutamate receptors
TENS: Transcutaneous electrical nerve stimulation
CCI: Chronic constriction injury
EA: Electroacupuncture
NR2B: N-methyl-d-aspartate receptor type 2B
CFA: Complete Freund's adjuvant
GT: Glutamate transporter
CIP: Chronic inflammatory pain
PAG: Periaqueductal gray
RVM: Rostral ventromedial medulla
DRN: Dorsal reticular nucleus
5-HT: 5-Hydroxytryptamine
MOR: *μ* opioid receptor
LHb: Lateral habenula
GAT1: GABA transporter 1
5-HT1AR: 5-Hydroxytryptamine 1A receptors
SNL: Spinal nerve ligation
COX-1: Cyclooxygenase-1
PGs: Prostaglandins
TNFR1: TNF-*α* receptor 1
MAPK: Mitogen-activated protein kinase
ERK: Extracellular signal-regulated kinase
MA: Manual acupuncture
GPCRs: G protein-coupled receptors
JNK: c-Jun-N-terminal kinase
ACA: Acupuncture-combined anesthesia
CPIP: Chronic postischemic pain
TRPV1: Transient receptor potentia1 vanilloid 1
L5Tx: L5 transection
